# Analysis of a panel of antibodies to APC reveals consistent activity towards an unidentified protein

**DOI:** 10.1038/sj.bjc.6603873

**Published:** 2007-06-26

**Authors:** M L Davies, G T Roberts, N Stuart, J A Wakeman

**Affiliations:** 1 North West Cancer Research Fund Institute, School of Biological Sciences, University of Wales Bangor, Bangor LL57 2UW, UK

**Keywords:** adenomatous polyposis coli, APC antibody specificity, siRNA, colorectal cancer cells

## Abstract

Acquisition of truncating mutations in the adenomatous polyposis coli (APC) protein underlies the progression of the majority of sporadic and familial colorectal cancers. As such, the localisation patterns and interacting partners of APC have been extensively studied in a range of systems, relying on the use of a broad panel of antibodies. Until recently, antibodies to APC have been used largely unchecked. However, several recent reports have been invaluable in clarifying the use of a number of antibodies commonly used to detect APC. Here, we analyse the specificity of a further subset of antibodies to APC. We used a panel of six commercially available antibodies (directed to the amino and carboxy termini of APC) and confirm the detection of full-length APC by immunoblotting. We demonstrate that a 150 kDa protein, also reproducibly detected by this panel of antibodies, is unlikely to be APC. We present data for the immunological staining patterns of the APC antibodies and validate the results through RNAi. Using this approach, we confirm that the apical staining pattern, observed by immunofluorescence and previously reported in cell systems, is unlikely to be APC. Finally, we present our data as a summary of APC-antibody specificities for APC.

Adenomatous polyposis coli (APC) is a large multifunctional tumour suppressor protein that has critical functions in both development and the normal functioning of adult cells (Joslyn *et al*, 1991; Kinzler *et al*, 1991; Miyoshi *et al*, 1992). Germ-line mutations in APC cause the disease known as familial adenomatous polyposis, where the affected individual accumulates hundreds of polyps in the colon during the second and third decades of life (Polakis, 1997). Such a condition predisposes the sufferer to colorectal carcinomas. Somatic mutations in APC underlie the majority of cases of sporadic colorectal cancers. The majority of mutations occurring in sporadic tumours are found in an area known as the mutation cluster region (MCR) corresponding to amino acids 1284–1580 (Miyoshi *et al*, 1992). One critical tumour-suppressor function of APC is thought to reside in the sequences downstream of the MCR. Truncating mutations in APC result in an inability to downregulate the activity of the oncogene *β*-catenin, allowing activation of wnt-signaling pathway targets (Fodde *et al*, 2001).

Localisation studies of proteins are important indicators of function. Full-length APC has been shown to be localised along the lateral plasma membrane in mouse epithelial cells (Miyashiro *et al*, 1995) and, more recently, in polarised cultured epithelial cells (Rosin-Arbesfeld *et al*, 2001). The latter localisation was shown to be dependent on an intact actin cytoskeletal network. Localisation of full-length APC is also very prominent in basal most regions, where it is present in clusters, dependent on intact microtubules (Rosin-Arbesfeld *et al*, 2001; Mogensen *et al*, 2002). A recent study showed that both full-length and truncated APC are present in the cytoplasm and that previous contradicting reports had been the result of aberrant antibody specificities. (Brocardo *et al*, 2005). The latter report (amongst others) highlights the importance of confirming the detection specificities of antibodies to APC.

In addition to full-length protein, many different forms of APC are known to exist within a cell as a result of alternative splicing, post-translational modifications and degradation (reviewed by Polakis, 1997). Functionally distinct pools of APC have been reported with the suggestion that some pools may have a function other than degradation of *β*-catenin (Reinacher-Schick and Gumbiner, 2001; Penman *et al*, 2005). In general, two distinct pools of intracellular APC are envisaged, one microtubule-bound and found at membrane protrusions and the other plasma membrane bound in an actin-dependent manner (Nathke *et al*, 1996; Rosin-Arbesfeld *et al*, 2001; Mogensen *et al*, 2002). A large multifunctional protein such as APC is an excellent candidate for the existence of functional variants with different roles in various tissues, stages of development and physiological states.

Furthermore, a number of studies have found APC to be localised apically with a range of independent antibodies directed to both the amino and carboxy terminus of APC in a variety of different cell lines and human and mouse tissues, including normal colon and colorectal tumours (Miyashiro *et al*, 1995; Senda *et al*, 1996; Midgley *et al*, 1997; Reinacher-Schick and Gumbiner 2001; Anderson *et al*, 2002; Umar *et al*, 2005) (see Table 1b).

We therefore set out initially to analyse a protein band (150 kDa), which was detected by a panel of antibodies directed to the N and C termini of APC on Western blots of cell lysates from a number of colorectal cancer cell lines. As, for any cell line, it is always desirable to use at least two different antibodies to validate data, these Western blot data might have appeared to be convincing. However, on further analysis, it seemed unlikely that this protein was APC. We report these findings and present a summary of the antibody specificities for the panel of commonly used antibodies to APC. This study supports and expands on the recent study by Brocardo *et al* (2005) and includes commonly used antibodies to APC that were not tested in their study. The relevance of reporting such antibody specificities lies in the central importance of APC to cancer development.

## MATERIALS AND METHODS

### Cell lines

SW480 cells (ECACC, and gift from MRC laboratories, Leicester, UK) were grown in DMEM/10%FBS, HCT116 cells (ECACC) were grown in McCoy's 5a medium/10%FBS, HEK293 cells (ECACC) were grown in MEM/10%FBS. All cells were grown at 37°C with 5% CO_2_.

### Immunoprecipitation and Western blot

Cells for immunoprecipitation (IP) and Western blot were collected by scraping, resuspended in IP buffer (50 mM Tris-HCl (pH 7.4), 200 mM KAc, 0.5% Triton-X-100, 1 mM AEBSF, 10 *μ*M E-64, 2 *μ*g ml^−1^ aprotinin, 1 *μ*M pepstatin, 10 *μ*M bestatin, 100 *μ*M leupeptin, 1 mM sodium ortho-vanadate) and lysed in a FastPrep FP120. Lysates were then centrifuged at 20 000 **g** at 4°C for 20 min. For IP, supernatants were incubated with 20 *μ*g ml^−1^ antibody or normal IgG at 4°C for 2–3 h. Protein G linked to agarose beads was pre-blocked for 1 h in 3% milk and incubated with lysates for 1 h. Protein G beads were then washed three times in IP buffer. Immunoprecipitates were run on 7.5% SDS–PAGE gels and transferred to PVDF membrane in 2 × Towbin buffer with 0.02% SDS or CAPS with 10% methanol at 600 mA overnight. For whole-cell lysate, Western blots Laemmli buffer was added to whole-cell lysates, immediately following lysis and centrifugation and SDS—PAGE, and transfer carried out as above. Blots were probed with antibodies as follows: APC(Ab2), APC(Ab5), APC(Ab6) 1 : 100 (Calbiochem, Nottingham, UK); APC(H-290) 1 : 2000 (Santa-Cruz Biotechnology Inc., Heidelberg, Germany); APC(Ab120) 1 : 2500 (AbCam, Cambridge, UK); *α*Tubulin 1 : 10 000 (Sigma, Dorset, UK). Bands were quantified using QuantityOne (BioRad Laboratories Ltd., Hertfordshire, UK).

### Immunofluorescence

Cells were grown to confluence in 40 mm tissue culture dishes, or in on glass coverslips in 24-well plates for RNAi. Cells were fixed in 4% paraformaldehyde for 20 min, permeabilised with 0.2% Triton-X-100 for 15 min and blocked in PBS-5% FBS for 1 h at room temperature. Cells were incubated with primary antibodies Ab4 1 : 50 (Oncogene), Ab5 1 : 50, Ab6 1 : 50 (Oncogene), Ab120 1 : 100 (AbCam), Ali12–28 1 : 250 (Millipore, Watford, UK), *β*-catenin (E-5) 1 : 100 (Santa-Cruz) for 30–60 min at 37°C then washed three times in PBS. Cells were then incubated in secondary antibody (AlexaFluor 488 or 568 anti-mouse 1 : 400 (Molecular Probes, Paisley, UK), AlexaFluor 488 or 568 anti-rabbit) for 30–60 min at 37°C and again washed three times in PBS. Cells were imaged using a Carl Zeiss Axioplan2 LSM510 confocal microscope.

### RNAi

Cells were grown in six-well plates for Western blot and on glass coverslips in 24-well plates for immunofluorescence (IF). Cells were grown in normal media for 2 days then transfected with either APC siRNA or a non-interfering control using RNAifect (Qiagen, Crawley, UK). After approximately 16 h, cells were washed with PBS and fresh medium added. Cells were used for either IF or Western blot 48 h after transfection. The DNA target for APCe2 was 5′-TATGGCTTCTTCTGGACAGAT-3′ (within exon 2 of APC), APCc2 was 5′-AATCGCCTGAACTCCTTTATT-3′. The control non-interfering siRNA's DNA target sequence was 5′-AATTCTCCGAACGTGTCACGT-3′. As an additional negative control, an APC siRNA, which failed to reduce the levels of APC in the cells was used. The DNA target for this siRNA, APCc1, was 5′-GTCCTGTATCAGAGACTAAT-3′. APCc1 and APCc2 are within the region corresponding to the last 300 amino acids at the C terminus of APC. The Qiagen validated siRNA, Hs_APC_6, was also used; for this siRNA, HiPerFect (Qiagen) was used for transfection. Cells were grown in normal media for 24 h before transfection, and were used for Western blot and IF 48 h after transfection. Non-interfering siRNA controls were carried out alongside.

## RESULTS

### Detection of a 150-kDa immunoreactive protein in epithelial cells using a panel of antibodies to APC

**Figure 1:**
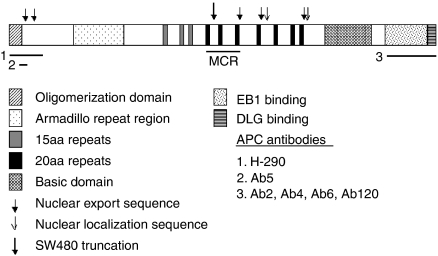
Positions of APC epitopes recognised by antibodies used in this study. H-290 was raised to a peptide corresponding to amino acids (aa) 2–289 at the N terminus of APC. Ab5 has been shown to bind to aa 60–73 (Kraus *et al*, 1996), which corresponds to exon 2 of APC. Ab2, Ab4, Ab6 and Ab120 have been raised to a peptide corresponding to the last 300 aa of APC.

**Figure 2:**
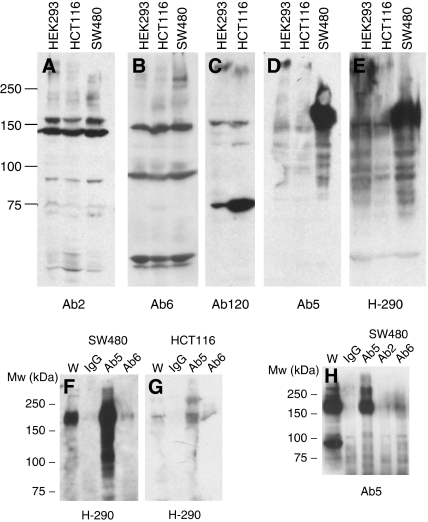
Multiple APC antibodies detect a band at 150 kDa on Western blot as well as detecting full-length and/or truncated APC. The N-terminal antibodies, Ab5 and H-290 (**D**,**E**) detect full-length, truncated APC in SW480 and an additional band at 150 kDa. The C-terminal antibodies detect full–length, but not truncated APC (**A**–**C**) and an additional band at 150 kDa. Immunoprecipitation and Western blot demonstrates that many of these antibodies are detecting the same protein at around 150 kDa (**F**–**H**). Full-length APC in HCT116 is not visible in (**G**), but could be seen in the lysate and IP lanes but not IgG control lane on longer exposures (data not shown). Although all these antibodies detect multiple bands in addition to APC, the band at 150 kDa is the only one that is recognised by multiple APC antibodies.

We used a panel of antibodies directed to the N and C termini of APC in Western blotting experiments on whole-cell extracts, to determine the size of APC present in a range of cultured cell lines and to confirm that the same protein sizes were detected by all the antibodies. The antibodies used, and the amino-acid regions to which they were raised are shown in Figure 1 and Table 1a, b. The cell lines HCT116 (colorectal cancer cell line) and HEK293 (Human embryonic kidney) both express full-length APC (314 kDa), whereas SW480 expresses a truncated APC, whose predicted size is 147 kDa, but actual running size is 152–155 kDa. Western analysis showed that a band representing full-length APC was detected at 314 kDa by all antibodies used (Ab5, H-290, Ab2, Ab6, Ab120) (Figure 2A-E). Ab4 fails to detect full-length APC on Western blot (data not shown); however, this antibody is not recommended by the manufacturer for use in immunoblotting. N-terminal antibodies detected truncated APC in SW480 cells at 150 kDa (Figure 2D,E). In addition, we also observed a further band that migrated to around 150 kDa, which was detected using all the antibodies directed to both the N and the C termini of APC (Figure 2A-E) (although this protein migrated to a size of 150 kDa, we estimate the actual size of this protein to be slightly less than this, as it is slightly smaller than the truncated APC expressed by SW480 cells whose predicted size is 147 kDa). That a 150 kDa protein was detected by C-terminal antibodies to APC is surprising, as SW480 has a mutation causing a truncation at 1338 amino acids, eliminating the expression of C-terminal sequences from full-length APC (We used two different sources of SW480 cells with the same result (data not shown). Furthermore, sequencing across the mutated region of genomic DNA derived from SW480 cells revealed that the expected mutation was present (data not shown)).

We then carried out IP and Western blot with a number of APC antibodies to test whether different antibodies would detect the same 150-kDa protein. Both N- and C-terminal antibodies were used for IP and products from the IP reactions were then immunoblotted against Ab5 or H-290 (N-terminal) antibodies. In all lanes containing antibody to APC following Western blotting, a band was observed at 150 kDa, suggesting that the C- and N-terminal APC antibodies tested each recognise the same species of 150 kDa (Figure 2F-H). Immunoprecipitation and Western blot with N-terminal antibodies in SW480 gave a band at 150 kDa, which was the truncated APC expressed in this cell line (Figure 2F,H). Immunoprecipitation with both N- and C-terminal antibodies and Western blot in HCT116 gave a band at the expected size for full-length APC on longer exposures (data not shown). In total, for the immunoblotting analysis, we used four independently derived monoclonal antibodies to APC (Ab2, Ab5, Ab6 and Ab120) and one polyclonal antibody to APC (H-290).

### Immunofluorescence staining patterns of APC-antibodies Ab5, Ab4, Ab6 and Ab120

**Figure 3:**
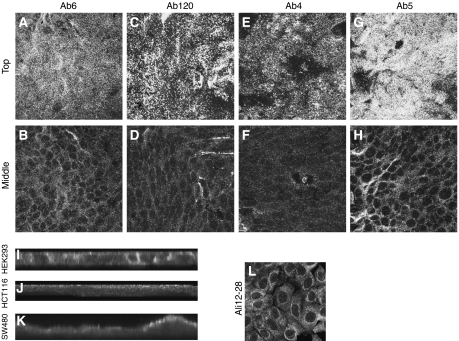
APC antibodies Ab6, Ab120, Ab4 (C-terminal) and Ab5 (N-terminal) all detect an apical protein in HCT116 (**A**,**C**,**E**,**G**), with some more faint cytoplasmic staining (**B**,**D**,**F**,**H**). Ab4 shows localisation to the apical, lateral and occasionally basal membranes in HEK293, but gives an apical localisation with more faint cytoplasmic staining in the colorectal cancer cell lines HCT116 (**J**) and SW480 (**K**). (**I**,**J**,**K**) show side views of the cells (projection of confocal Z-stack). Ali12-28 gives a cytoplasmic localisation (**L**).

After many conflicting reports, it has recently been shown (through a combination of Western blotting, siRNA and IF) that endogenous full-length and truncated APC is predominantly located in the cytoplasm (Brocardo *et al*, 2005). Our panel of antibodies included commonly used antibodies to APC that were not tested in the study by Brocardo and co-workers. We used confocal microscopy and IF localisation with our panel of N- and C-terminal APC antibodies to determine the localisation of protein detected by each antibody. Confocal microscopy on confluent HCT116 cells using the N-terminal-directed antibody to APC, Ab5 showed predominant apical staining with fainter cytoplasmic staining (Figure 3G,H). Similar studies using the C-terminal-directed antibodies, Ab4, Ab6 and Ab120 demonstrate the same staining pattern, that is, apical localisation, with fainter cytoplasmic staining (Figure 3A–F). We also carried out IF in the cell lines SW480 (colon carcinoma) (Figure 3K) and HEK293 cells (human embryonic kidney) (Figure 3I). Again, IF was carried out using the panel of N- and C-terminal-directed APC antibodies: Ab4 (Figure 3I–K), Ab5, Ab6 and Ab120 (data not shown). Confocal microscopy demonstrates that, in confluent HCT116 and SW480 cells, staining is predominantly localised to the apical plasma membrane with faint cytoplasmic staining (Figure 3J,K). However, in confluent HEK293 cells, in addition to the apical membrane staining, we also observed lateral membrane staining, and occasionally, some cells were observed with staining in basal membrane regions (Figure 3I).

Three of the antibodies, which detected apical protein, Ab5, Ab6 and Ab120, also detected the 150-kDa protein on a Western blot. Ab4 detected apical protein, but failed to detect the 150-kDa protein or full-length APC by Western blot. H-290 did not detect apical protein on IF, but did detect the 150-kDa protein. Ab2 did not give any staining above background levels on IF, but did detect the 150-kDa protein on a Western blot.

The staining patterns observed using these antibodies are not in accordance with the observations of Brocardo *et al* (2005). Furthermore, that the C-terminal APC antibodies should give the same staining pattern as the N-terminal antibodies to APC in SW480 cells was unusual, as C-terminal APC sequences should not be expressed in these cells. We therefore followed this up with siRNA to APC to determine the specificity of these antibodies to APC. In addition, to confirm that we could detect cytoplasmic APC using an antibody shown to be specific for APC, we also used the antibody Ali12-28 for IF in all the cell lines. Using this antibody, we were able to confirm that this showed cytoplasmic staining as expected (Figure 3L).

### siRNA to confirm the specificity of the antibodies

siRNAs were designed to exon 2 and to a region within the last 300 amino acids of the C terminus of APC. A commercially available and validated siRNA to APC, Hs_APC_6 (Qiagen), was also used. Transient transfection of the different siRNAs was carried out to allow knockdown of gene expression for an optimised time period of 48 h in SW480 and HCT116 cells. Specific siRNA knockdown of protein detected by antibodies to APC was compared to cells treated with a non-interfering RNA and to untreated cells.

**Figure 4:**
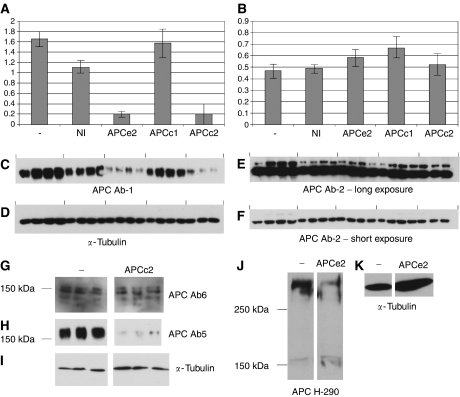
RNAi for APC does not reduce the levels of the 150-kDa protein. APC siRNAs APCe2 and APCc2 reduce levels of truncated APC in SW480 (**A**, **C**), but not the 150-kDa protein detected by Ab2 (**B**, **E**) or the nonspecific band below the 150 kDa (**F**). Negative controls shown are NI (a non-interfering control siRNA) and APCc1 (an siRNA designed to APC which failed to reduce APC levels). Tubulin loading control is shown in (**D**). Graphs (**A**, **B**) show normalised data for the Western blots shown below (**C**–**F**). The 150-kDa band detected by Ab6 (C-terminal) in SW480 resolves into several bands on 5% SDS–PAGE, none of these bands are downregulated by APC RNAi (**G**), Western blot with Ab5 (N-terminal) confirmed that APC had been successfully downregulated (**H**), loading control is shown in (**I**). Full-length APC, but not the 150-kDa protein, was downregulated by APC siRNA in HCT116 (**J**), tubulin loading control shown in (**K**).

Whole-cell lysates were prepared from SW480 cells and the products run on a gel and immunoblotted using antibodies that would detect only the 150-kDa protein (C-terminal-directed antibodies), and antibodies that would detect both truncated APC and the 150-kDa protein (N-terminal-directed antibodies). Three to four repeats were carried out for each siRNA. Both the siRNA directed to exon 2, APCe2, and one of the siRNAs directed to the last 300 amino acids of APC, APCc2, were shown to be able to knock down truncated APC in SW480 (Figure 4A,C). One of the siRNAs designed to the last 300 amino acids of APC, APCc1, failed to knock down APC and was used as an additional negative control in these experiments. Ab2, a C-terminal antibody, detects two proteins of around 150 kDa in SW480 and other cell lines, the larger of which appears to be the same size as the 150 kDa bands detected by other APC antibodies. Neither of these proteins was knocked down by APC siRNA directed to exon 2 and the last 300 amino acids of APC, under the conditions used here (Figure 4B,E,F).

When SW480 lysates were run on 5% SDS–PAGE and immunoblotted against the APC antibody, Ab6 (a C-terminal antibody), the band detected at 150 kDa was resolved into several bands (Figure 4G). None of these bands were downregulated by APC siRNA under the conditions used here. Western blot with Ab5, an N-terminal antibody which detects the truncated APC expressed in SW480 cells, confirmed that the RNAi was successful (Figure 4H).

RNAi with an APC siRNA directed to exon 2 of APC in HCT116 failed to knock down the 150-kDa protein detected by H-290, an N-terminal antibody, under the conditions used here. Full-length APC was successfully knocked down in these cells (Figure 4J).

**Figure 5:**
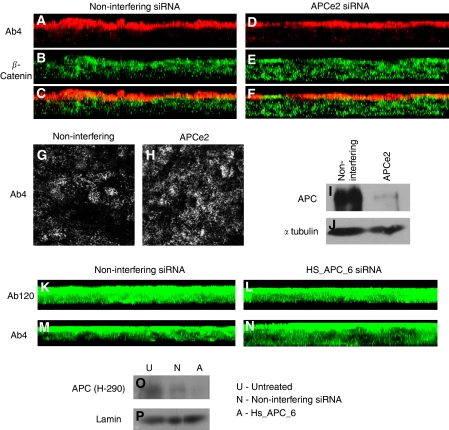
No reduction in staining was seen with Ab4 after RNAi for APC with APCe2 siRNA (**A**–**H**). An apical slice from a confocal Z-stack is shown in (**G**, **H**) and a projection of a confocal Z-stack (side view of cells) is shown in (**A**–**F**). Western blot with the APC antibody Ali12–28 shows the RNAi successfully reduces APC levels (**I**), loading control shown in (**J**). Normalised reduction was approximately fivefold. The APC siRNA Hs_APC_6 fails to reduce the apical staining seen with the APC antibodies Ab4 (**K**, **L**) and Ab120 (**M**, **N**). Western blot confirmed that APC levels were reduced in RNAi treated cells (**O**), loading control shown in (**P**).

To test whether the apical protein detected by many of these antibodies could be knocked down by siRNAs directed to APC, RNAi and IF staining was carried out in HCT116 cells. Both side views (projection of a confocal Z-stack) (panels A–F, K–N) and an apical slice (panels G,H) are shown in Figure 5. No reduction in apical staining in cells transfected with two different APC siRNAs was seen with both APC antibodies, Ab4 and Ab120, under the conditions used here. To confirm successful knockdown of APC in HCT116 cell lysates from the same batch of cells, lysates were immunoblotted against the APC antibody Ali12-28 (Figure 5I) or H-290 (Figure 5O). This showed successful knockdown of APC.

### APC antibody specificity summary

Table 1a, b is presented as a summary table, showing data for the specificity of the APC antibodies in this and other studies. We summarise their activity in (i) Western analysis (detection of a 314 and 150 kDa band) and (ii) their corresponding immunostaining patterns (if the antibody is suitable for both applications). We have also presented published data from other laboratories to provide a comprehensive summary table of APC antibody specificities.

## DISCUSSION

The specific detection of APC is of paramount importance due to its central role as a tumour suppressor protein, and its ability to result in the development of carcinomas when mutated. Here, we describe the detection specificities of a panel of antibodies that have been developed to detect APC. This work extends several recent publications in this area and importantly serves to provide a comprehensive analysis of APC antibody specificities (Roberts *et al*, 2003; Brocardo *et al*, 2005).

Initially, we carried out Western blotting experiments, using a panel of antibodies to APC to confirm the size of expressed APC in a number of different cell lines. We observed a band at approximately 150 kDa with a number of antibodies in the three different cell lines used. One possible explanation for the presence of this band was expression of an unusual and cryptic splicing event in exon 15 of APC. This 150-kDa protein could not have been a post-translational modification, as it was detected by C-terminal-directed antibodies to APC, beyond the termination codon in SW480, in addition to it being detected with N-terminal antibodies. We then carried out further experiments to confirm or refute this based on validating the specificities of the antibodies used to detect APC, as an alternative explanation for the presence of this 150-kDa band on APC Western blots could be that several antibodies were crossreacting with proteins of approximately the same size.

Two approaches were followed to confirm the specificity of antibodies through (i) reciprocal co-IP and blotting experiments and (ii) RNAi experiments. The former approach suggested the presence of a 150-kDa cryptic splice variant of APC, or that several APC antibodies were all crossreacting with the same 150-kDa protein. The latter approach suggested that the antibodies used were likely to be detecting a protein other than APC at 150 kDa, as this 150-kDa protein could not be knocked down by APC RNAi. The apical protein seen on IF staining with several APC antibodies also failed to be knocked down by APC RNAi.

We considered the possibility that a cryptic splicing event produces a variant that is more stable than ‘conventional’ APC. The region of APC between amino acids 1034 and 1337 is responsible for the ubiquitin-proteasome-mediated downregulation of APC (Choi *et al*, 2004). This region also encompasses the MCR and the truncating mutation in SW480. It remains a feasible proposition that a cryptic splicing event at the start of exon 15 may give rise to a form of APC that has this region spliced out, thereby, becoming more stable and therefore not ‘knocked down’ under the conditions used here. However, RT–PCR with primers to exons 1,2 and 3 of APC and two primers within the sequence corresponding to the last 300 amino acids at the C terminus of APC, successfully amplified full-length APC, but did not give any products which would account for a spliced form of APC 150 kDa in size (data not shown). Northern blot gave a band at the expected size for full-length APC and no smaller bands (data not shown). As no alternatively spliced APC mRNA could be detected by either of these methods, it seems unlikely that the 150-kDa protein we observed could be an alternatively spliced form of APC, which is more stable than the full-length or truncated APC proteins expressed in the cell lines used in the RNAi experiments (Brocardo *et al* (2005) looked for C-terminal splice variants of APC by Western blotting, but did not detect bands corresponding to potential splice variants. One antibody used in their studies, Ab4, is not recommended for use in Western blotting and fails to detect full-length APC (Table 1a, b), the two other C-terminal antibodies used are not listed in our report).

Our findings with this panel of antibodies led us to further clarify and present a summary of the specificity of the panel of antibodies to APC that were used in this study. Many of these antibodies are able to reliably detect full-length APC and/or truncated APC. Of the antibodies tested by us, only Ab4 and N-15 failed to detect full-length or truncated APC. However, all the antibodies tested, which were able to detect APC, also gave several nonspecific bands. Five out of all the antibodies studied detect an apical protein by IF; APC RNAi confirmed that this protein is not likely to be APC and therefore it appears that these antibodies cannot reliably detect conventional APC by IF staining. Validation of the specificity of these antibodies will serve to lessen ambiguities in the literature.

## Figures and Tables

**Table 1a:** Summary of APC antibodies that detect apical protein and/or 150-kDa protein

**Antibody**	**Clone no. / ID no.**	**Epitope**	**Localisation**	**Western blot**
APC (Ab2)	IE1	C-term 300 aa	—	Full length, 150 kDa
APC (Ab4)	HG2	C-term 300 aa	Apical/cytoplasmic	Fails to detect APC
APC (Ab5)	CF11	N-term 60–73 aa	Apical/cytoplasmic^a^	Full length, truncated, 150 kDa
APC (Ab6)	DB1	C-term 300 aa	Apical/cytoplasmic	Full length, 150 kDa
APC (Ab120)	c-APC 28.9	C-term 300 aa	Apical/cytoplasmic	Full length, 150 kDa
APC (H-290)	H-290	N-term 1–289 aa	Cytoplasmic	Full length, truncated, 150 kDa
APC (N-15)	N-15	N-term 2–16 aa	Apical/cytoplasmic	Fails to detect APC

a We did note some batch variation with this antibody, with not all batches detecting apical protein.

**Table 1b:** Summary of other APC antibodies reported to detect apical staining

**Antibody**	**Reference**	**Epitope**	**Apical staining**
APC-C-ter	Miyashiro *et al* (1995)	C-terminal 14 aa	Apical staining in mouse duodenum, proximal and distal colon
APC-C-ter	Senda *et al* (1996)	C-terminal 14 aa	Lateral and apical cytoplasm in mouse colon
APC 3122	Midgley *et al* (1997)	N-terminal 8–347 aa	Apical staining in a variety of human tissue sections
APC 3161	Midgley *et al* (1997)	C-terminal 2813–827 aa	Apical staining in a variety of human tissue sections
APC N-15	Reinacher-Schick and Gumbiner (2001)	N-terminal 2–16 aa	Apical staining in a variety of cell lines and mouse colon
Variety of N- and C-terminal APC antibodies	Anderson *et al* (2002)	—	Apical staining in normal colon, polyp and carcinoma with a variety of antibodies
APC Ab7	Umar *et al* (2005)	N-terminal 1–226 aa	Apical in transmissible murine colonic hyperplasia
APC Ob	Umar *et al* (2005)	N-terminal	Apical in transmissible murine colonic hyperplasia
APC F-3	Umar *et al* (2005)	N-terminal 2–289 aa	Apical in transmissible murine colonic hyperplasia, and weak apical staining at the base of normal crypts
